# Whole brain radiotherapy with radiosensitizer for brain metastases

**DOI:** 10.1186/1756-9966-28-1

**Published:** 2009-01-06

**Authors:** Gustavo Arruda Viani, Gustavo Borges Manta, Ellen Carrara Fonseca, Ligia Issa De fendi, Sergio Luis Afonso, Eduardo Jose Stefano

**Affiliations:** 1 Department of Radiation Oncology at Marilia Medicine School, São Paulo, Brazil; 2 Medical Student at Marilia Medicine School, São Paulo, Brazil; 3 Department of Ophthalmology at Marília medicine school, São Paulo, Brazil

## Abstract

**Purpose:**

To study the efficacy of whole brain radiotherapy (WBRT) with radiosensitizer in comparison with WBRT alone for patients with brain metastases in terms of overall survival, disease progression, response to treatment and adverse effects of treatment.

**Methods:**

A meta-analysis of randomized controlled trials (RCT) was performed in order to compare WBRT with radiosensitizer for brain metastases and WBRT alone. The MEDLINE, EMBASE, LILACS, and Cochrane Library databases, in addition to Trial registers, bibliographic databases, and recent issues of relevant journals were researched. Significant reports were reviewed by two reviewers independently.

**Results:**

A total of 8 RCTs, yielding 2317 patients were analyzed. Pooled results from this 8 RCTs of WBRT with radiosensitizer have not shown a meaningful improvement on overall survival compared to WBRT alone OR = 1.03 (95% CI0.84–1.25, p = 0.77). Also, there was no difference in local brain tumor response OR = 0.8(95% CI 0.5 – 1.03) and brain tumor progression (OR = 1.11, 95% CI 0.9 – 1.3) when the two arms were compared.

**Conclusion:**

Our data show that WBRT with the following radiosentizers (ionidamine, metronidazole, misonodazole, motexafin gadolinium, BUdr, efaproxiral, thalidomide), have not improved significatively the overall survival, local control and tumor response compared to WBRT alone for brain metastases. However, 2 of them, motexafin- gadolinium and efaproxiral have been shown in recent publications (lung and breast) to have positive action in lung and breast carcinoma brain metastases in association with WBRT.

## Background

Brain metastases represent a sizeable health care problem. An estimated 20–40% of cancer patients will develop multiple brain metastases [1], and 30–40% will develop a single metastasis [2] during the course of their illness. Therapeutical approaches to brain metastases include surgery, whole brain radiotherapy (WBRT), stereotactic radiosurgery (SRS), and chemotherapy. Treatment decisions must take into account clinical prognostic factors in order to maximize survival and neurological function whilst avoiding unnecessary treatments [3-11]. Radiosensitizers are chemical or pharmacologic agents that increase the lethal effects of radiation if administered with it. In an attempt to improve outcomes, studies have examined the use of whole brain radiotherapy combined to radiosensitizers [12-18]. There are many chemicals capable of rendering cells or tissue more sensitive to radiation, but it only those drugs for which there is a differential response between the tumor and dose-limiting normal tissue that may be of benefit radiotherapy. Dozens of clinical trials have been performed, most of which have been inconclusive or have shown results with a borderline results [19-27]. Tsao et al. has presented the results of five randomized controlled trials [5,19-23] that examined the use of radiosensitizers in addition to WBRT. However, none of those trials detected a benefit in terms of overall survival or brain response (CR + PR). Moreover, this meta-analysis did not evaluate the incidence of adverse effects, the differences on quality of life or the neurocognitive progression. Since its publication, other studies have been published, investigating new radiosensitizers. So, the aim of our meta-analysis is to evaluate the outcomes and adverse effects of the randomized clinical trials in the treatment of cerebral metastases using radiosensitizer combined to WBRT.

## Methods

### Objectives

The aim of this study is to analyze the efficacy of whole brain radiotherapy plus radiosensitizer compared to whole brain alone for patients with brain metastases in terms of overall survival, disease progression, response to treatment and adverse effects of treatment. Secondary objective was to investigate the treatment effect on neurological status and quality of life.

Criteria for considering studies for this review

### Types of studies

All randomised and quasi-randomised controlled trials were eligible for inclusion.

### Types of participants

Adult patients were eligible if they had TC or MRI-demonstrated brain metastases from histologically proven solid tumors, required WBRT, with any Karnofsky performance status and RPA class with brain metastases originated from solid tumors, excluding small-cell lung cancer, germ cell tumors, and lymphomas. There were no restrictions regarding gender or nationality. Trials of prophylactic whole brain radiotherapy in which whole brain radiotherapy was used with no evidence of existing brain metastases were excluded. Studies that examined the use of surgery or whole brain radiotherapy, or both, for single brain metastases were also excluded

### Types of intervention

All trials were included where adult patients were randomly assigned to receive WBRT given in daily fractions, with or without radiosensitizer.

### Types of outcome measures

Data for the following outcome measures were analyzed:

The overall survival in six months. Intracranial progression-free duration was defined as the time from randomization or entry to the trial until progressive brain disease is diagnosed. Local brain response was considered as the percentage of patients achieved complete response (CR) or partial response (PR) to treatment. Complete response was defined as complete radiographic disappearance of brain metastases. Partial response was defined as more than 50% decrease in size of the brain metastases on CT or MR imaging. Local brain control was reported to as the percentage of patients with unchanged or improved serial post-treatment CT or MRI scans judged either as a complete response (CR), partial response (PR), or stable disease (SD), with improving or stable neurological symptoms or neurological examination results. SD is defined as a 0 to 50% decrease in size of all lesions with stabilization neurological symptoms or neurological examination results and stable dexamethasone dose. Progressive disease is defined as an increase in the size of any lesion, the development of new lesions, or a decrease in neurological symptoms or examination requiring an increase in dexamethasone dose. Quality of life, symptom control and neurological function assessed by any scale.

### Research strategy for identification of studies

Medline and manual research was done (completed independently and in duplicate) to identify all published (manuscripts and abstracts) randomized controlled trials (RCTs) that comparing WBRT plus radiosensitizer treatment for brain metastases to WBRT alone. The Medline research was done on PubMed between 1966 and 2008 with no language restrictions, using the search terms "brain metastases", "radiotherapy" or "metastases," "whole brain radiotherapy" or "radiosensitizer" and "brain radiation." The second research was done through CancerLit, EMBASE, LILACS and the Cochrane Library to identify randomized trials published between January 1998 and July 2007, using MeSH headings (brain metastases, whole brain radiotherapy, radiosensitizer/sc {Secondary}, ex-lode Clinical Trials, clinical trial {publication type}) and text words (brain, cancer, radiotherapy:, radiosensitizer, trial, and study) without language restrictions. All the researched abstracts were screened by relevance. Manual research was done by reviewing articles and abstracts cited in the reference lists of identified RCTs, by reviewing the first author's article, abstract file, from reference lists of retrieved papers, textbooks and review articles. Also, abstracts published in the Proceedings of the Annual Meetings of the American Society of Clinical Oncology were systematically researched for evidence relevant to this meta- analysis. The selection of studies for inclusion was carried out independently by two of the authors (V-GA and S- EJ). Each study was evaluated for quality using the scale of 1 to 5 proposed by Jadad [18]. When reviewers disagreed on the quality scores, discrepancies were identified and a consensus was reached. Trial data abstraction was also done independently and in duplicate, but abstractors were not blinded to the trials' authors or institution. Any discrepancies in data abstraction were examined further and resolved by consensus.

### Data analysis

The proportion of patients surviving at six months was treated as dichotomous data. This was estimated from Kaplan-Meier probability curves of survival at six months. For forest plot analyses, mortality data (the inverse of survival at six months) was plotted. An odds ratio (OR) less than 1.0 indicated that the patients in the experimental treatment group experienced fewer deaths compared to those in the control group. Intracranial progression-free duration was defined as the period during which there was no intracranial tumor growth and no new brain metastases. This was treated as continuous data. The heterogeneity of instruments used and the differences in reporting quality of life, symptom control, and adverse effects outcomes were described and not pooled.

## Results

**Figure 1:**
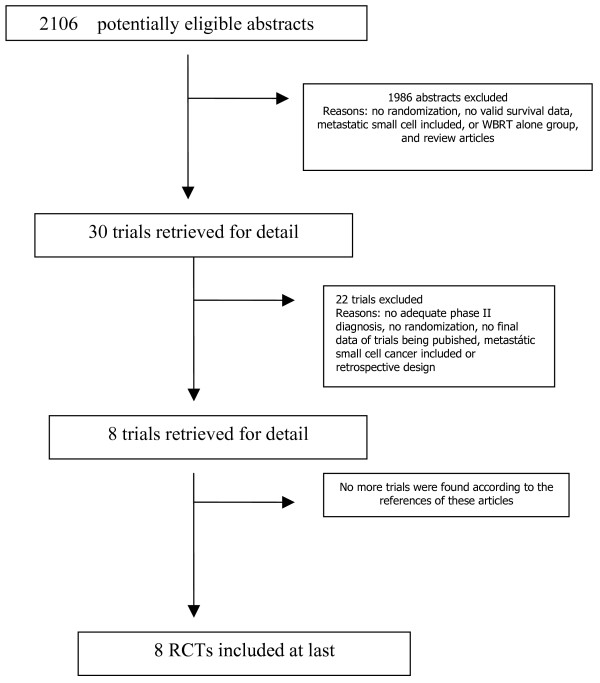
**Flowchart according to QUOROM statement criteria**.

The electronic and manual research revealed 2016 entries. These were screened and 38 full text articles were retrieved for further assessment. We excluded 30 studies, as they were either not randomized studies or were not comparisons of medical versus surgical treatment. The reasons for exclusion are detailed in the excluded studies figure 1.

Eight fully published trials [19-26] examined the use of radiosensitizers in addition to whole brain radiotherapy (2217 patients in total). The radiosensitizers used were lonidamide [19], metronidazole [20], misonidazole [21], bromodeoxyuridine (BrdU) [22], and motexafin gadolinium [23,24], efaproxiral [25] and thalidomide [26]. Mehta et al [23] reported on survival and neurological outcomes. A follow-up report by Meyers et al. [27] reported specifically on neurocognitive outcomes from the group of patients randomized in the motexafin gadolium trial by Mehta et al [25] reported on the use of whole brain radiotherapy and supplemental oxygen with or without RSR13 (efaproxiral), a novelty in radiation sensitizer that performs as a modifier of hemoglobin to facilitate oxygen release. Table 1 describes the characteristics of the studies included in this meta-analysis.

**Table 1:** Randomized studies of WBRT and radiosensitizers versus WBRT alone

Study	Study arms	No. of pts randomized	Overall median survival	Overall survival at 6 months	Response (CR + PR)
**DeAngelis (19)**	3000 cGy/10 fr + lonidamine	31	4.0 m	NR	37%
	3000 cGy/10 fr	27	5.4 m		55%
**Eyre (20)**	3000 cGy/10 fr + metronidazole	57	2.8 m	14	27%
	3000 cGy/10 fr	54	3.2 m	13	24%
**RTOG-7916(21)**	3000 cGy/6 fr + misonidazole	220	3.1 m	68	NR
	3000 cGy/6 fr	216	4.1 m	83	
	3000 cGy/10 fr + misonidazole	211	3.9 m	65	
	3000 cGy/10 fr	212	4.5 m	72	
**Mehta(23)**	3000 cGy/10 fr + MGd	193	5.2 m	82	NR
	3000 cGy/10 fr	208	4.9 m	85	
**RTOG-8905(22)**	3750 cGy/15 fr + BrdUrd	35	4.3 m	12	63%
	3750 cGy/15 fr	37	6.12 m	20	50%
**REACH (25)**	3000 cGy/10 fr + RSR13	265	5.4 m	119	48%
	3000 cGy/10 fr	250	4.4 m	96	36%
**RTOG- 0118(26)**	3750cGy/15 fr + thalidomide	90			NR
	3750 cGy/15 fr	93			
**SMART(24)**	3000 cGy/10 fr + MGd	279	NR	NR	NR
	3000 cGy/10 fr	275			

### Setting and participants

The radiosensitizers studied were lonidamide, metronidazole, misonidazole, motexafin gadolinium, bromodeoxyuridine (BrdU), RSR13 (efaproxiral) and thalidomide. In regards to the outcomes of interest, none of the trials reported on either proportion of patients who were able to reduce their daily dexamethasone dose or duration of reduced dexamethasone requirements. All trials used WBRT with total dose range 30 – 37.5 Gy in 10–15 fractions.

### Overall survival at six months

**Figure 2:**
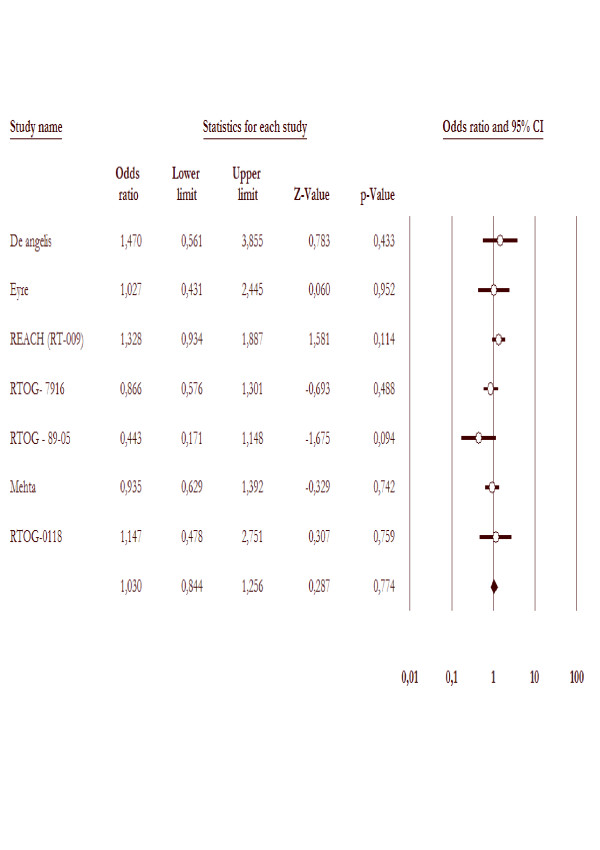
**Overall mortality in the trials included in this meta-analysis comparing WBRT with radisensitizer to WBRT alone**.

Seven studies reported overall survival as one of the outcomes. Altogether, the analyses included 7 trials with 1763 patients. The overall mortality rates were not decreased for WBRT with radiosensitizer arm (517/878 = 58.8%) compared to WBRT alone arms (519/885 = 58.6%). The test for heterogeneity was not statistically significant with p value 0.28. The overall odds ratio suggests that there is no difference between WBRT with radiosensitizer arms and WBRT alone arms in terms of overall mortality rate with p value 0.77, as demonstrated in figure 2.

### Local brain tumor response

**Figure 3:**
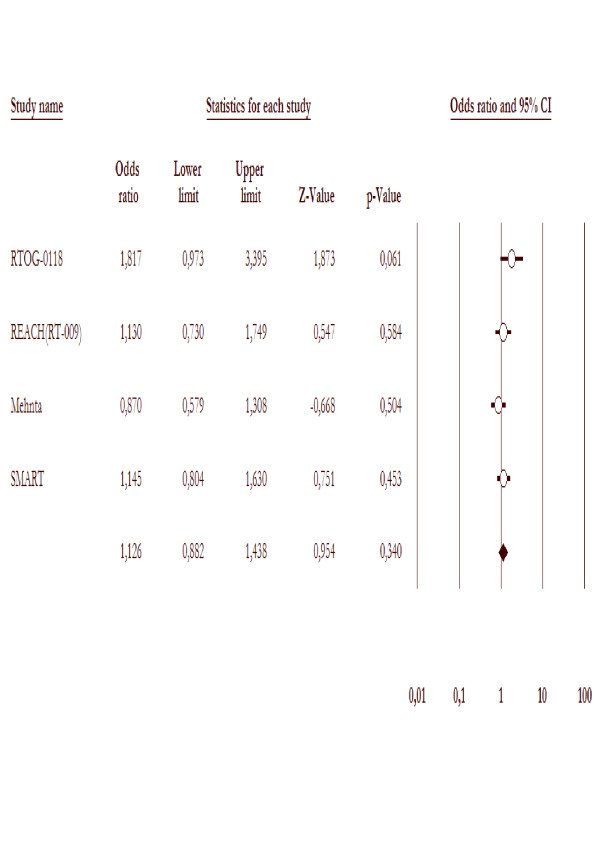
**Local brain tumor response in the trials included in this meta-analysis comparing WBRT with radiosensitizer to WBRT alone**.

Four trials [19,20,22,25] reported on local brain tumor response rates (either complete response (CR) or partial response (PR)). There was no significant difference (P value 0.98) in response rate among those patients receiving only whole brain radiotherapy (135/548 = 24.6%) and those receiving treatment with whole brain radiotherapy and radiosensitizers (135/548 = 24.6%), OR = 0.8(95% CI 0.5 – 1.03), as figure 3.

### Central nervous system progression

**Figure 4:**
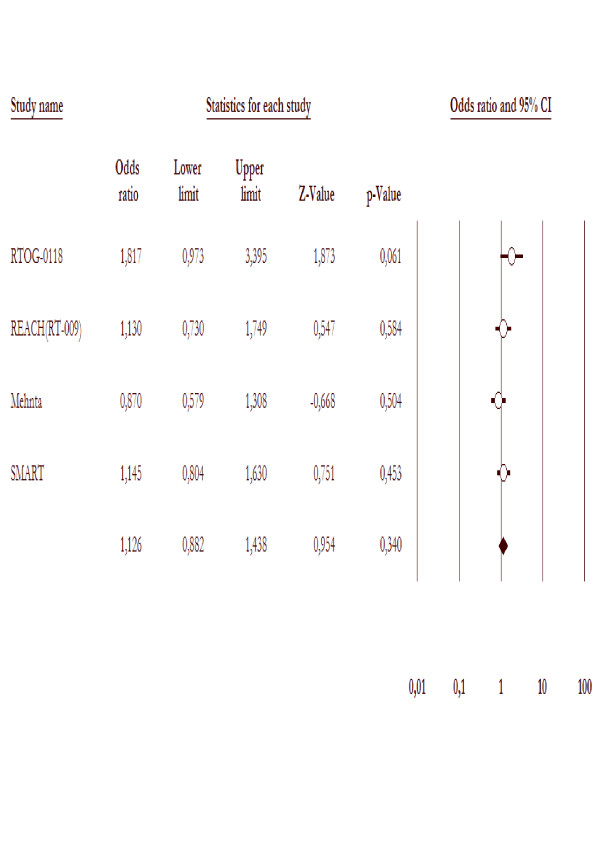
**CNS progression in the trial included in this meta-analysis comparing WBRT with radiosensitizer to WBRT alone**.

Four studies [19,20,22,25] had reported CNS progression data (three published and one in abstract form), 1099 patients were included in the analysis. There were no more CNS progression in WBRT alone (150/551 = 27.2%) compared to WBRT with radiosensitizer (135/548 = 24.6%). The likelihood of CNS progression was 1.1-fold higher (95% CI 0.8 – 1.4) in WBRT arms. Test for heterogeneity was not significant with p value of 0.15, as is in the figure 4.

### Quality of life and the neurocognitive progression

Three trials [25,27,28] reported quality of life outcomes. In the REACH trial, the numbers and percentages of patients with stable or improving quality of life, were assessed by the Spitzer Questionnaire (SQI) and KPS at 1, 3, and 6 months after WBRT. A larger percentage of patients in the efaproxiral arm had stable or improving quality of life scores over the course of the follow-up visit. In a subgroup analysis, Suh et al. showed that in breast cancer patients the quality of life was improved in the WBRT plus efaproxiral arm compared to the WBRT alone arm (*P *< 0.019). Meyers et al., evaluating patients of the Mehta et al trial, reported no significant difference in time to progression of brain-specific quality of life (FACT-BR) measures in any of the treatment groups. There was also no statistically significant difference between treatment arms in time to neurocognitive progression on the patients treated for whole brain radiotherapy with or without motexafin gadolinium. Patients with lung cancer (but not other types of cancer) who were treated with motexafin gadolinium in addition to whole brain radiotherapy tended to have improved memory and executive function (P value 0.062) and improved neurological function. In the RTOG-0118, quality of life was measured by the SQLI and the Folstein MMSE was used to determine neurocognitive progression. SQLI and MMSE were administered at baseline and at 2-month intervals. MMSE was scored with a threshold value associated with neurocognitive functioning (absolute cutoff level of 23) and with the use of corrections for age and educational level. In a secondary analysis of 156 patients neurocognitive and quality of life outcomes were examined and Corn et al. [27] demonstrated that in spite of the neurocognitive decrease, QOL remained stable during treatment and follow-up, and poor neurocognitive function may predict clinical deterioration.

### Adverse effects

All seven published studies that assessed the addition of radiosensitizers to whole brain radiotherapy reported serious adverse effects. In the REACH trial, most of the treatment-emergent adverse effects were grade 1 (mild) to grade 2 (moderate) in severity in both treatment arms. The most commonly reported grade 3 adverse effects in efaproxiral-treated patients were hypoxemia, which was reported in 11% of patients (29 out of 266 patients). In the RTOG 0118 [26], most of the experienced toxicities were not severe but they were significant enough to limit compliance with protocol therapy. The rate of patients experiencing Grade 3–4 treatment-related adverse events on the thalidomide arm (39/84) was significantly higher than the rate on the WBRT arm (11/92) (p < 0.0001). In the SMART trial [24], published by Mehta et al. in abstract form only, most common adverse effects were skin discoloration (66%), urine discoloration (35%), nausea (27%), fatigue (21%) and hypertension (18%). However, grade 3–4 toxicity was very rare 1–4%. DeAngelis et al. [19] found that the most common side effects of lonidamide and WBRT were myalgia (68%), testicular pain (42%), anorexia (26%), ototoxicity (26%), malaise or fatigue (26%), and nausea and vomiting (19%). In the Eyre study [20] it was reported 51% incidence of nausea and vomiting compared to 3.2% in the whole brain radiotherapy arm alone. Komarnicky et al. [19] showed that the administration of the misonidazole with WBRT was well tolerated and produced no grade-three neurotoxicity or ototoxicity. Phillips et al. [22], in the RTOG 8905, reported three fatal toxicities in 34 patients randomized to whole brain radiotherapy with administration of the radiosensitizer BrdU. One death resulted from a severe Stevens-Johnson skin reaction and two other deaths were due to neutropenia and infection. Mehta et al. reported grade three and four adverse events: hypotension (5.8%), asthenia (2.6%), hyponatremia (2.1%), leukopenia (2.1%), hyperglycemia (1.6%), and vomiting (1.6%) in the 193 patients randomized to the whole brain radiotherapy and motexafin gadolinium arm.

## Discussion

In most patients with brain metastasis, WBRT is the mainstay of treatment and efforts to improve the outcome of WBRT continue. These efforts include radiation sensitizers such as efaproxiral, motexafin gadolinium, and thalidomide.

Historically, chemical modifiers of radiation effect have had little impact on overall average survival times in human trials of brain metastases. Misonidazole, bromodeoxyuridine (BUdR), lonidamine, nimustine, fluorouracil, and others have failed to show significant benefit in randomized trials [19-26]. Recent developments suggest a new interest in this approach with three compounds that show as a promise as radiosensitizers: motexafin gadolinium, thalidomide and efaproxaril.

Efaproxaril is a small, synthetic molecule that non-covalently binds to hemoglobin and decreases its oxygen binding affinity and shifts the oxygen dissociation curve to the left, increasing p50 and tissue pO2. It exerts its effects based on an increase in tumor oxygen levels, thereby circumventing restrictions due to the blood brain barrier [14,28-30] Shaw et al [14] conducted a phase II, open-label, multicenter study of efaproxaril plus WBRT in 57 patients with brain metastases. The results were retrospectively compared to the RTOG RPA brain metastases database; the average survival time for the efaproxaril treated patients was 6.4 months compared to 4.1 months for the database (*P *< .0174).

Motexafin-gadolinium (MGd) is a metalloporphyrin redox modulator that demonstrates selective tumor localization and catalyzes the oxidation of a number of intracellular metabolites, such as ascorbate, glutathione, and nicotinamide adenine dinucleotide phosphate, thereby generating reactive oxygen species, and depleting the pools of reducing agents necessary to repair cytotoxic damage [31]. Preliminary studies in patients with brain metastases treated with MGd and WBRT demonstrated radiological responses in 68% to 72% of patients [31].

Thalidomide inhibits the angiogenic activity of bFGF (FGF2), a peptide with pleiotropic activities that performs on various cell types, including endothelial cells, following interaction with heparan-sulfate proteoglycans and tyrosine kinase FGF receptors [32-34]. FGF2 seems to stimulate both tumor cell growth and angiogenesis through paracrine mechanisms [33]. Thalidomide can improve blood flow through tumor neovasculature, resulting in improved oxygenation and decreased interstitial fluid pressures [34]. Improved tumor oxygenation during WBRT would improve the therapeutical ratio for the use of radiation for tumors with hypoxic cells. Thalidomide was being given as salvage therapy for recurrent gliomas, and a Phase II trial documented that cranial radiation therapy could be delivered with concomitant thalidomide administration without unusual toxicity [35].

The presence of hypoxia in solid tumors has been acknowledged for over 50 years. Hypoxic cells are more resistant to standard chemotherapy and radiotherapy, in addition to being more invasive and metastatic, resistant to apoptosis, and genetically unstable [36]. Thus, it is not surprising that hypoxia has been considered an attractive target for the development of new anti-cancer therapies, including pro-drugs activated by hypoxia, hypoxia-specific gene therapy, targeting the hypoxia-inducible factor 1 transcription factor, and recombinant anaerobic bacteria [38]. The potential to improve local control and survival by hypoxia modification was demonstrated by a meta-analysis of 83 clinical trials [38] and a number of therapeutical strategies have also been established to overcome tumor hypoxia by improving oxygen supply either by oxygen or carbogen breathing or by increasing the hemoglobin level and oxygen delivery [39,40].

Unfortunately, our data, including 7 RCTs with 1.763 patients, using these three promise radiosensitizers combined with WBRT, have not shown a sizeable increase in average survival (5.2 *v *4.9 months; *P *= 0.48), CNS progression or local brain tumor response. (9.5 *v *8.3 months; *P *= 0.95). None of those trials detected any benefit for theses end point mentioned above. In the trial by Mehta et al. [23], no difference in survival or time to neurological progression was seen in the use of motexafin gadolinium and WBRT versus WBRT alone. However, a subgroup analysis, carried out for lung cancer patients was reported to as an improvement in neurological progression favoring the motexafin gadolinium and WBRT arm. The results for the lung cancer subgroup can only be interpreted as a hypothesis generated as there was no a priori decision to analyze this group independently. On the basis of these results, a phase III trial was conducted exclusively in patients with NSCLC; a preliminary report was presented at the 2006 ASCO meeting. In this international trial, 554 patients were randomly assigned to WBRT (30 Gy in 10 fractions) plus MGd (5 mg/kg with each RT treatment) or WBRT alone [24]. There was a trend to an increased time to neurological progression, the primary endpoint in the study, in patients receiving MGd (15.4 versus 10 months with RT alone). In another large RCT study [27], Suh et al. showed in a subset analysis that the addition of efaproxiral to WBRT reduced the death rate by 46% (*P *< 0.0086). Quality of life was improved in the WBRT with efaproxiral arm compared to the WBRT alone arm (*P *= 0.019). Quality-adjusted survival was statistically and significantly improved by the addition of efaproxiral to WBRT (*P *= 0.001).

Patients with brain metastasis may suffer a certain degree of neurocognitive function (NCF) impairment from multiple factors including the tumor, WBRT, neurosurgical procedures, chemotherapy and other neurotoxic therapies (including anticonvulsants and steroids), or from paraneoplastic effects induced by the malignancy [41].

Three trials included in this meta-analysis evaluated neurocognitive function. However, we were not able to pool these data, due to the different methods used for this outcome. In addition to that, studies involving NCF deterioration should be carefully interpreted. NCF decline in the literature is often defined statistically and there is little consensus as to the actual clinical relevance of a statistical definition. Conventionally, the measures used, such as the Folstein mini-mental status examination, are rather crude, and it is crucial to develop sensitive and practical neurocognitive function testing to characterize these changes [30]. In particular, the sensitivity of mini-mental status examination has been shown to be problematical in detecting subtle neurocognitive dysfunction in patients with brain metastasis where clinically apparent WBRT-induced dementia is rare (1.9–5.1%) [42,43]. All of these factors can potentially affect the manifestation of changes in neurocognition in a patient with newly developed brain metastases.

## Conclusion

Our results show that WBRT with radiosensitizer have not improved the overall survival, local control and tumor response compared to WBRT alone for brain metastases. Despite the use of WBRT with radiosensitizer, outcomes are poor and efforts should be made to incorporate multimodality approaches including surgery and radiosurgery to improve survival. In spite of this apparent negative result, radiosensitizers may be helpful in specific subsets of patients with brain metastases from lung and breast cancers. This can lead to a superior therapeutical ratio by enhancing the benefit derived from whole brain radiotherapy resulting in an improvement of neurocognitive decrease, neurological progression, and quality of life.

## Competing interests

The authors declare that they have no competing interests.

## Authors' contributions

GAV conceived of the study, done the statistical analysis and wrote the manuscript. GBM collected the RCTs and patient's clinical data. ECF, LIF, SLA and EJS participated in the design of the study and helped write the paper. All authors read and approved the final manuscript.
